# An estimation of the financial consequences of reducing pig aggression

**DOI:** 10.1371/journal.pone.0250556

**Published:** 2021-05-05

**Authors:** Rachel S. E. Peden, Simon P. Turner, Irene Camerlink, Faical Akaichi

**Affiliations:** 1 School of Natural and Environmental Sciences, Newcastle University, Newcastle, United Kingdom; 2 Animal Behaviour & Welfare, Animal and Veterinary Sciences Research Group, Scotland’s Rural College (SRUC), Edinburgh, United Kingdom; 3 Institute of Genetics and Animal Biotechnology, Polish Academy of Sciences, Jastrzebiec, Poland; 4 Department of Rural Economy, Environment and Society, Scotland’s Rural College, Edinburgh, United Kingdom; Universidade do Porto Instituto de Biologia Molecular e Celular, PORTUGAL

## Abstract

Animal welfare scientists have accumulated knowledge and developed interventions to improve livestock welfare, but these are poorly adopted in commercial practice. Animal welfare interventions are rarely tested for economic viability and this limits their uptake. This study employs Stochastic Partial Budgeting (SPB) to determine the viability of animal welfare improvements. Aggression between pigs is used as an example because there is a large literature base from which to draw interventions, and the problem has persisted for decades without resolution. Costs and benefits of three interventions to control aggression (pre-weaning socialisation, synthetic maternal pheromones and large social groups) were estimated by reviewing the academic and industry literature and by conducting a survey of sixteen pig farmers. The net effects were compared to farmers’ willingness to pay (WTP) for interventions to reduce aggression as identified by recent research. Results are consistent with prior research which indicates that improving animal welfare generally comes at a cost to producers. Nevertheless, pre-weaning socialisation resulted in a neutral or positive net effect 38% of the time and should be central to campaigns promoting the control of aggression in the industry. Exposing pigs to synthetic maternal pheromones did not improve profitability but the net costs were small and within the realms of WTP for a sub-group of farmers with animal welfare goals. The net costs of converting existing buildings in order to house pigs in large social groups were beyond the realms of farmers’ WTP. The approach adopted in this study, of combining SPB with WTP from the sector, should be extended to other animal welfare issues.

## Introduction

Animal welfare scientists continue to accumulate useful knowledge identifying specific changes to animal management, nutrition and genetics which can improve the welfare of animals in agriculture. However, these interventions are poorly adopted in commercial practice (e.g. [1, 2]). In this paper, we focus on the case study of aggressive behaviour between pigs. A wealth of research has identified effective solutions to this problem but these are not adopted on most farms. Here we explore the economic consequences of aggression mitigation strategies to identify which, if any, are financially viable. We argue that cost-benefit analysis is required to assess the viability of solutions to many animal welfare issues.

Although certain animal welfare standards are required to ensure productivity (e.g. feed, housing, health), in other cases measures to promote animal welfare can be more expensive than the returns gained [3–6]. Farmers are heterogenous in their willingness to adopt and pay for animal welfare interventions beyond the minimum required by legislation; and their decisions are determined by range of factors, including the economic value derived from returns in productivity (i.e ‘use values’) and the value of improved animal welfare independent of any returns in productivity (i.e. ‘non-use values’: [7–9]. Although farmers’ decisions are not solely influenced by financial returns, they must largely be confident that the costs will be covered by improvements in productivity [10–12].

Despite general knowledge that for farmers the cost price of production is important, only a handful of studies have modelled the economic feasibility of animal welfare interventions. Specifically, researchers have estimated the economic feasibility of animal welfare improvements in broiler [13, 14] and laying hen production [13, 15], in cattle production [16], in extensive sheep production [17] and in intensive pig production [6, 13, 18]. Overall, these studies reveal that improving animal welfare comes at a cost to producers. Thus, in order to initiate a change in practice it is essential that researchers identify practical and economically viable solutions.

This study utilises Stochastic Partial Budgetting (SPB) in order to determine the viability of animal welfare improvements. Furthermore, the net effects were compared to farmers’ willingness to pay (WTP) as identified by prior research [19]. Aggression between pigs is used as an example because there is a large literature base from which to draw solutions and the problem has persisted for decades without resolution [2]. Specifically, aggression between pigs is common in commercial farming as unfamiliar pigs are regularly regrouped, and this results in high levels of physical aggression as animals establish dominance relationships [20]. Aggression is a significant threat to animal welfare, with several negative outcomes. These are: injuries mainly in the form of skin lesions [21] and lameness [22]; exhaustion and physical fatigue [23]; restricted access to limited resources such feeders, drinkers and preferred lying areas [24]; and stress which can have negative, transient effects on the immune system [25], reproduction [26, 27] and growth performance [28, 29]. A large body of peer reviewed literature has identified some specific changes to pig management, nutrition and genetics which can reduce the occurrence or intensity of aggression at regrouping (review articles: [2, 30]). However, aggression research has had little impact on commercial practice, and aggression remains an important animal welfare issue [2]. It is possible that misalignment between the focus of pig aggression research and financial constraints on farmers has resulted in impractical or unaffordable solutions.

This study aims to determine the viability of the most promising aggression control strategies. The economic consequences of investing in three aggression control strategies are estimated. The choice of aggression control strategies included in this study is justified in the section ‘Pig management scenarios’. Cost and benefits of the interventions were estimated relative to a control situation (e.g., no change). A key risk when estimating the costs and benefits of interventions is their over/underestimation. In order to minimise this risk a combined approach was employed whereby the costs and benefits of each pig management scenario were estimated by conducting a survey of pig farmers to gain information on the finances in practice (see section ‘Estimation of costs and benefits: farmer survey’); by reviewing the academic and industry literature (see section ‘Estimation of costs and benefits: the literature’); and by conducting sensitivity analysis using SPB (see section ‘Stochastic partial budgeting model’). The survey was not used to facilitate the estimation of costs associated with exposing pigs to synthetic maternal pheromones as these were estimated using commercially available product information. The final SPB model is presented in Section ‘Results’.

## Materials and methods

### Pig management scenarios

Aggression control strategies were chosen by employing a stepwise selection process which: 1) identified only effective aggression control strategies as indicated by the greatest consensus in the literature that they effectively reduce aggression (e.g. literature reviews: [2, 30]; 2) identified only strategies which could be realistically implemented by farmers under commercial farming conditions (e.g. where they are dependent upon bought-in products, those products are commercially available); and 3) identified only strategies which could be employed to manage aggression on commercial units housing growing / finishing pigs intended for slaughter. Although pigs are most commonly mixed at weaning [31], this study focussed on controlling aggression between growing / finishing pigs because farmers perceive aggression as a greater problem between growers / finishers than between weaners, and would be more likely to adopt a solution for these pigs [32]. Moreover, aggression at regrouping of growing / finishing pigs has a greater risk of impacting upon growth performance, and more risk of lameness and injuries than at weaning when pigs are lighter and weaker [33]. All aggression control strategies which did not meet the above criteria were eliminated from the analysis. Three pig management scenarios met this criteria, these were:

1. **Pre-weaning socialisation of piglets.** This is the most studied aggression control strategy and there is strong evidence that this social experience results in reduced aggression at weaning [34] and in the grower stage [33] under both experimental [35] and commercial conditions [36]. Specifically, two litters of piglets are allowed to mix from the second week of life, when piglets would start to encounter other litters under natural conditions [37], and remain together until weaning. It is presumed to reduce aggression by allowing piglets to learn social skills which permit more rapid formation of stable dominance relationships in later social encounters [33, 34, 38]. This study assumed the socialisation of pairs of litters in conventional farrowing systems, whereby lactating sows are housed with their piglets in individual farrowing crates. In these systems, pre-weaning socialisation requires the modification or removal of barriers between adjacent farrowing pens.
2. **Housing pigs in relatively large social groups.** This strategy results in reduced aggressive behaviour [39–41] likely reflecting the adoption of a less aggressive social strategy due to the higher number of potential competitors [39]. Thus, the benefits of establishing dominance relationships with pigs that will be met relatively infrequently do not justify the costs of establishing those relationships in the first place. Furthermore, prior experience of large social groups leads to less aggression at future regroupings [39–41]. The optimum group size for the control of aggression has never been established, and is likely to vary between farms depending on buildings and feeding regimes [42, 43]. Nevertheless, the group size for growing / finishing pigs must be sufficiently large (more than 12 individuals) to have an impact on aggression levels [40], and much larger groups (>80 pigs) are more effective [39, 41]. This study assumed the formation of groups of 100 or more pigs as this is a realistic number that is already achieved by a subgroup of farmers in practice [44]. Furthermore, this group size is biologically relevant as it is likely to be sufficiently large to prevent pigs establishing dominance relationships with all group members. This study assumed the conversion of existing buildings by removing fencing between adjacent pens and by making necessary changes to feeders and drinkers. The costs of building new units suitable for large social groups was not considered in the current study.
3. **Exposing pigs to synthetic maternal pheromones.** This strategy reduces the frequency of fights at mixing in breeding sows [45], adult pigs [46], and weaners under experimental [47] and commercial farming conditions [48]. Synthetic pheromones contain several fatty acids similar in composition to pig appeasing pheromone, which is released naturally through sow skin secretions in order to regulate nursing behaviours [49]. The number of skin lesions as a result of aggression is reduced up to seven days following mixing in groups exposed to the synthetic pheromone, suggesting that it does not merely postpone the occurrence of aggression but results in the more rapid formation of stable social relationships [48]. The pheromone is commercially available to buy in odour diffusers, and this study assumed the application of diffusers in accordance with product application instructions.

### Estimation of costs and benefits: Farmer survey

Sixteen farmers completed the paper based survey during January 2019 after piloting with two researchers and one pig farmer. Informed consent was obtained for all participants and the survey received ethical approval from the Human Ethical Review Committee at the University of Edinburgh. All responses were confidential and could not be linked to any individual. No controversial questions were asked and there were no possibilities for causing distress or embarrassment to participants. The full survey is available in S1 File. All analyses were conducted in SPSS (version 25) and Microsoft Office Excel (2016).

Farmers (n = 16) completed the survey during one farmer discussion group (n = 8) or through contact with colleagues at Scotland’s Rural College whilst on farm visits (n = 8). Farmers were given no prior notice that they would be asked to participate in this study; therefore bias was unlikely to be introduced due to interest in the study topic. The response rate was 100%. The target population was UK pig farmers who kept pigs at the growing and/or finishing stages of production. We chose to recruit participants face-to-face with a paper-based survey in order to maintain quality of responses and due to poor response rates of farmers to previous online surveys [44, 50]. Farmers had on average 30 years of experience working with pigs (std 16.0, range 6–65), fifteen were male (and one unspecified), and all were based in Scotland. Their farms were assured by the Royal Society for the Prevention of Cruelty to Animals (RSPCA) (n = 4), the Scottish Society for the Prevention of Cruelty to Animals (ScottishSPCA) (n = 7), Quality Meat Scotland (n = 16) and Red Tractor (n = 2). All farmers housed their growing and finishing pigs indoors and farm size varied widely (see Table 1) as is representative of intensive pig farms in the UK [51, 52]. All farmers regrouped unfamiliar pigs at weaning, two regrouped at the grower stage, and five regrouped at finishing. The average group size for growing / finishing pigs was 64 (std 24.9, range 15–400). Five farmers indicated that they already allow litters to co-mingle prior to weaning on their farm, and two indicated that they use chemical additives to feed, water or air (e.g. appeasing pheromones, citronella sprays, herbal remedies). Responses were weighted equally regardless of prior use of interventions. Farmers estimated that it costs them roughly £118.13 (std £14.38, range £100 - £135) to produce each pig, and this is roughly consistent with national production costs as calculated by AHDB Pork [53]. Limitations of the current sample are acknowledged in Dicussion section titled ‘Strengths and Limitations’.

**Table 1:** Mean number of pigs kept at each stage of production, standard deviation and range.

Stage of production (n)	Mean number on farm	Standard deviation	Range
Weaners (15)	1359	1014.5	70–4050
Growers (11)	999	790.8	70–2600
Finishers (16)	1447	994.2	70–2800
Sows (15)	390	181.0	90–800

Number of farmers to keep pigs at each stage of production is specified in parentheses.

In order to estimate the costs of allowing pairs of litters to mix prior to weaning and changing existing structures in order to house pigs in large social groups (100+ pigs), farmers were asked to list the changes needed on their unit in order to implement both strategies and to estimate the monetary value of initial investment and on-going costs. Farmers were instructed to answer in hindsight if relevant. For example, those who already mix pairs of litters prior to weaning were asked to list the costs that they encountered when they made the change. If this had always been their method of production, they were asked to leave the question blank. In order to ensure that responses were interpreted correctly, farmers were asked to make it clear how costs were described (e.g. per pig, per pen or per entire farm). In order to make farmers’ responses comparable, all values reported were converted to reflect the costs ‘per pig produced’. Detailed information on how farmers’ responses were converted into the ‘per pig produced’ scale are available in the ‘S2 File’. The various investment and on-going costs likely to arise when implementing each aggression control strategy, as estimated by farmers, can be seen in Table 2. Seven farmers stated that the labour requirements for removing barriers between adjacent farrowing pens would result in zero costs. This was linked to it taking an insignificant amount of time for farmers’ to remove barriers themselves, and that they did not regard this use of their own time as a cost.

**Table 2:** Farmers’ survey responses regarding the estimation of initial investment costs and on-going costs likely to arise when implementing each aggression control strategy.

Pig management scenario	Initial investment cost (n farmers to specify)	Mean estimate of monetary cost (n farmers to provide a monetary estimate, range)	On-going cost (n farmers to specify)	Mean estimate of monetary cost (n farmers to provide a monetary estimate, range)	
**Pre-weaning socialisation**	Modify walls between adjacent farrowing crates to make the removal of barriers possible (e.g. make gaps/ doors in the wall) (n = 2)	£0.03 (n = 2, £0.01- £0.05)	Labour requirements for removing barriers between adjacent farrowing pens when litters are 2 weeks old (n = 10)	£0.05 (n = 10, £0.00 - £0.50)	
	No initial investments/ changes (n = 8)	£0.00 (n = 8, N/A)	Increased mortality (n = 1)	£0.22 (n = 1, N/A)	
**Housing growing/ finishing pigs in groups of 100 or more**	Remove pen divisions (n = 8)	£0.04 (n = 6, £0.01- £0.14)	Labour requirements (n = 9) (e.g. for more time to pick out fat pigs for slaughter)	£0.67 (n = 5, range £0.04-£1.04)	
	Re-design penning (n = 1)	£0.08 (n = 1, N/A)	Lower performance (n = 1)		No monetary estimate provided
	Move feeders (n = 1)	£0.06 (n = 1, N/A)	Increased mortality (n = 1)		No monetary estimate provided
			No on-going cost (n = 2)		£0.00 (n = 2, N/A)

Costs are described ‘per pig produced’.

The total mean costs of implementing each pig management scenario as estimated by farmers can be seen in Table 3. The costs of increased mortality as predicted by farmers (Table 2) were excluded because increased incidence of mortality was not supported by peer reviewed research (see Section 4 ‘The literature’). Only farmers who fully answered the questions regarding the monetary estimation of both initial investment costs *and* on-going costs were included in these analyses. For allowing litters to mix prior to weaning, n = 8 farmers fully answered the questions; for housing pigs in large social groups, n = 6 farmers fully answered the questions.

**Table 3:** Mean (standard deviation, range) initial investment cost, on-going cost and total cost of implementing each pig management scenario as estimated by farmers.

Pig management scenario	Mean initial investment cost (std, range)	Mean on-going cost (std, range)	Mean total cost (std, range)
**Pre-weaning socialisation**	£0.02 (£0.03, £0.00 - £0.06)	£0.06 (£0.18, £0.00 - £0.50)	£0.08 (£0.17, £0.00 - £0.50)
**Housing pigs in large social groups (100+)**	£0.05 (£0.06, £0.02 - £0.14)	£0.56 (£0.51, £0.00 - £1.04)	£0.61 (£0.51, £0.02 - £1.15)

Costs are described ‘per pig produced’.

In order to facilitate the estimation of monetary benefits, farmers were asked to indicate which positive outcomes, from a list provided, that they would expect to encounter if they saw a 50% reduction in aggression on their farm. The results are presented in Table 4. Farmers were given the option to list any further benefits which were not included but none did so. Farmers were also asked to estimate how much money they would expect to save if aggression was reduced by 50% on their farm. Ten farmers provided an estimate, with a mean value of £0.30 per pig produced (std £0.55, min £0.00 –max £1.50). The extent to which aggression is reduced following implementation of an aggression control strategy varies widely in the literature. A figure of 50% was chosen for the purpose of this exercise, however this reflects the optimistic, upper-limit of achievable aggression reduction, and scaling for uncertainty over benefit scenarios was conducted in the economic model.

**Table 4:** The number of farmers to tick each positive outcome in response to the question ‘*please imagine that you see a reduction in regrouping aggression of 50% in your growers / finishers*. What benefits would you expect to see?’.

Positive outcome	Number of farmers to tick
**Improved growth rates**	12
**Reduced skin lesions/injury**	14
**Reduced labour requirements**	8
**Easier animal handling**	6
**Improved feed efficiency**	10
**Reduced veterinary costs**	10
**Improved job satisfaction**	10
**Reduced mortality**	10
**Higher sale weight**	1

Fourteen farmers responded to this question.

### Estimation of costs and benefits: The literature

A wide range of key performance indicators have been investigated in the academic and industry literature (see Table 5). Studies which tested these strategies and measured key performance traits were identified in a recent review of the aggression literature [2] and by searching Web of Science using the following search terms: ‘Pig’, ‘Sow’, ‘Aggression’.

**Table 5:** Results of a review of the academic and industry literature investigating the economic costs and benefits likely to arise when implementing each aggression control strategy in practice.

Pig management scenario	Economics costs	Economic benefits
**Pre-weaning socialisation**	**Cross-suckling** Whilst some studies found that cross-suckling does occur [35, 54, 55], others did not [34, 38]. Nevertheless, there is evidence that even where cross suckling is common it does not reduce the overall milk intake of the piglets [54] and there is no penalty in pre-weaning growth rate.	**Improved growth performance** Socialisation does not affect pre-weaning growth rate [34, 36], and can improve weaner performance by increasing growth rate following weaning [56, 57]. However, by 11 weeks old there are no differences in growth performance between socialised and control pigs [33]; therefore farmers would be unlikely to benefit from greater slaughter weights in socialised pigs.
	**Heightened mortality** Increased mortality was observed in multi-suckling systems where more than two litters were simultaneously co-mingled [58], however it has not been observed when pairs of litters are socialised [33, 34]	
	**Teat injury** Increased incidence of teat injury have been observed in sows of socialised litters and this could cause teats to become non-functional; thus, reducing the number of piglets the sow can nurse [33]. Nevertheless, the risks of teat injury can be minimised by considering the circumstances, such as sow and litter health, udder condition, litter size, and differences in age between neighbouring litters [33].	
**Housing pigs in large social groups (100+ pigs)**	**Increased labour costs** It is likely to take more time to catch individual pigs for treatment (e.g. vaccinations or tagging) [43].	**Reduced labour costs** The reduced requirement for gates and partitions in large groups reduces time taken to clean pens [43].
	**Reduced growth performance** There is evidence that growth performance is compromised in large groups of weaners and growers but not finishers [59].	**Reduced construction costs** The reduced requirement for gates and partitions would reduce construction costs [43, 60]; however this economic benefit would only apply to farmers building new units.
**Exposure to synthetic maternal pheromones**	**Purchase of diffusers** Diffusers are commercially available to buy on the market.	**Improved growth performance** Pheromones have improved growth performance and feed efficiency after weaning [47], however this is not supported by later work [48] and there is no evidence that growth performance is improved in growing / finishing pigs.
	**Increased labour costs** Labour requirements for installation of diffusers into pens according to product instructions.	

The literature indicated that cross-suckling and heightened mortality were unlikely to occur when allowing pairs of litters to socialise prior to weaning (Table 5). Although there is increased risk of sow teat injury when litters are socialised, this risk can be minimised through proper management of the intervention [33]. Therefore, the monetary costs of allowing litters to mix prior to weaning as estimated by farmers were included in the economic model with no additional penalties added (Table 6).

**Table 6:** The costs of each pig management scenario as included in the stochastic partial budgeting.

Pig management scenario		Costs (£) per pig produced		
	Source of cost	Minimum	Mean	Maximum
**Pre-weaning socialisation**	Modifying walls, increased labour requirements (as estimated by farmers)	**£0.00**	**£0.08**	**£0.50**
**Housing pigs in large social groups (100+ pigs)**	Re-organising pens, increased labour requirements (as estimated by farmers)	£0.02	£0.61	£1.15
	Lowered growth performance (as estimated based on literature)	£1.20	£1.60	£2.00
	**Total**	**£1.22**	**£2.21**	**£3.15**
**Pheromones**	Purchasing diffusers (as estimated based on market information)	£0.31	£0.39	£0.46
	Labour requirements for installing diffusers in pens (as estimated based on market information)	£0.01	£0.03	£0.04
	**Total**	**£0.32**	**£0.42**	**£0.50**

For housing pigs in large social groups (100+), the monetary costs as estimated by farmers were included in the economic model (Table 6). However, additional penalties were applied based on the peer-reviewed literature which indicated that average daily gain is reduced when housing growing, but not finishing, pigs in large social groups [59] (see Table 5). Although one farmer did anticipate lowered performance in large social groups, no monetary estimate was provided (Table 2). Therefore, these additional costs were estimated based on the findings of Turner et al. (2003) that average daily gain for a growing pig is 0.65 kg per day minus 0.00048 kg per additional pig. It was assumed that the average group size for growing pigs in the UK industry is 50. This assumption was based on a survey of a representative sample of 82 UK pig farmers which found that growing pigs were kept in groups of 47 on average (median 39, std 31.6, range 14–200) [19]. Thus, a penalty was added in order to account for lowered growth performance (see Table 6). Detailed information on how the costs of lowered growth performance were calculated is available in S3 File.

Synthetic maternal pheromones are commercially available in odour diffusers. Therefore, the costs of purchasing pheromones were estimated based on commercially available price information [61–63] and on product instructions which specified that diffusers should be placed 1.5m above the ground, one diffuser should be placed every 25m^2^, and the diffusers will release the pheromone for up to 6 weeks following opening. A further penalty was added in order to account for the increased labour required to install diffusers into pens (see Table 6). Detailed information on how the costs of purchasing diffusers and installing them in pens were estimated is available in S4 File.

The benefits of reducing aggression as estimated by farmers in the survey are consistent with the literature whereby the primary economic benefits of reducing aggression are likely to arise from alleviating injuries and stress caused by aggression (e.g. [20, 21]. Therefore, the monetary benefits of reducing aggression as estimated by farmers were used to develop the final economic model. Specifically, farmers’ estimated that a 50% reduction in aggression would save them on average £0.30 per pig produced and this was included as the ‘maximum’ benefit. This is because the extent to which aggression is reduced following implementation of an aggression control strategy varies widely in the literature, and a 50% reduction reflects the optimistic, upper-limit of achievable aggression reduction. The most likely value of £0.15 reflects a more expected reduction in aggression of 25% (£0.30 / 2). The minimum of £0.00 was the minimum benefit as estimated by farmers and reflects no monetary benefits as a result of reducing aggression (Table 7).

**Table 7:** The monetary benefits of each pig management scenario as included in the stochastic partial budgeting.

Pig management scenario		Benefits (£) per pig produced		
	Benefits	Minimum	Most likely	Maximum
**Pre-weaning socialisation**	Reduced aggression as estimated by farmers	**£0.00**	**£0.15**	**£0.30**
**Housing pigs in large social groups (100+ pigs)**	Reduced aggression as estimated by farmers	**£0.00**	**£0.15**	**£0.30**
	Reduced labour requirements when cleaning pens	£0.00	£0.01	£0.02
	**Total**	**£0.00**	**£0.16**	**£0.32**
**Pheromones**	Reduced aggression estimated by farmers	**£0.00**	**£0.15**	**£0.30**

The literature indicated no additional benefits for pre-weaning socialisation or exposing pigs to synthetic maternal pheromones. However, an additional benefit of housing pigs in large social groups was highlighted by the literature, whereby the reduced requirement for gates and partitions would reduce the time taken to clean pens between batches [43]. Therefore, an additional monetary benefit was added for this strategy. Detailed information on how this monetary benefit was estimated is available in S5 File.

### Stochastic partial budgeting model

Partial Budgeting (PB) is a financial tool that can support decision makers to assess the financial consequences of specific interventions, especially when detailed empirical data are unavailable. In particular, PB compares the benefits and costs resulting from implementing the proposed interventions, with respect to the current practice. Monetary units are used to quantify the costs and benefits and to provide a measure of the net benefit. The outcomes of different interventions can be compared to identify the most beneficial and, thus, the best allocation of limited resources [64]. PB has been widely applied across a variety of academic fields, including for animal health and welfare interventions [16, 65].

The PB uses a single-point value for each cost or benefit element. This renders the analysis deterministic and likely to be inaccurate due to the fact that costs and benefits of an animal welfare improvement vary largely across farms (e.g., due to variation in farm structure and size, variation in farm management approaches and workers’ skills). To account for the stochastic nature of the input variables for the partial budgeting model (e.g., quantities and expected costs and revenues), this study uses the stochastic partial budgeting (SPB) approach instead of the PB. In addition to estimating the average net effect of a change using single-point values (i.e. mean), the SPB allows the analyst to use a range of the estimated costs and revenues (e.g., minimum, mean, and maximum value) and attach to them probabilities of occurrence. A combination of the estimated values of costs and revenues and their probability distributions are used to determine the range and probability of the change’s final possible outcomes [66, 67]. For ease of presentation and interpretation, the final outcome of the stochastic analysis is generally graphed as a Cumulative Distribution Function (CDF).

The SPB was carried out as follows. First, the negative and the positive effect of implementing each of the three animal welfare interventions were computed based on the information displayed in Tables 6 and 7. For example, in the case of intervention “housing pigs in large social groups”, the negative effects arise from an increase in required labour and decrease in growth performance. By comparison, the positive effects involved a reduction in aggression and its negative consequences and a reduction in labour requirements when cleaning pens. Once all the negative and positive effects were obtained, the net effect of each animal welfare improvement was computed. The net effect was computed as the sum of the positive effect minus the sum of the negative effects. The intervention is said to be economically profitable if the calculated effect is positive.

Second, a stochastical analysis was carried out to account for the uncertainty associated with the estimated costs and revenues of the three interventions. The uncertainties around the estimated negative and positive effects were modelled by fitting triangular probability distributions. The triangular distributions are commonly used in stochastic analysis when the distribution of the estimated effects is difficult to determine, and the analyst only has two data points (i.e., minimum and maximum values) for each estimated effect.

**Fig 1:**
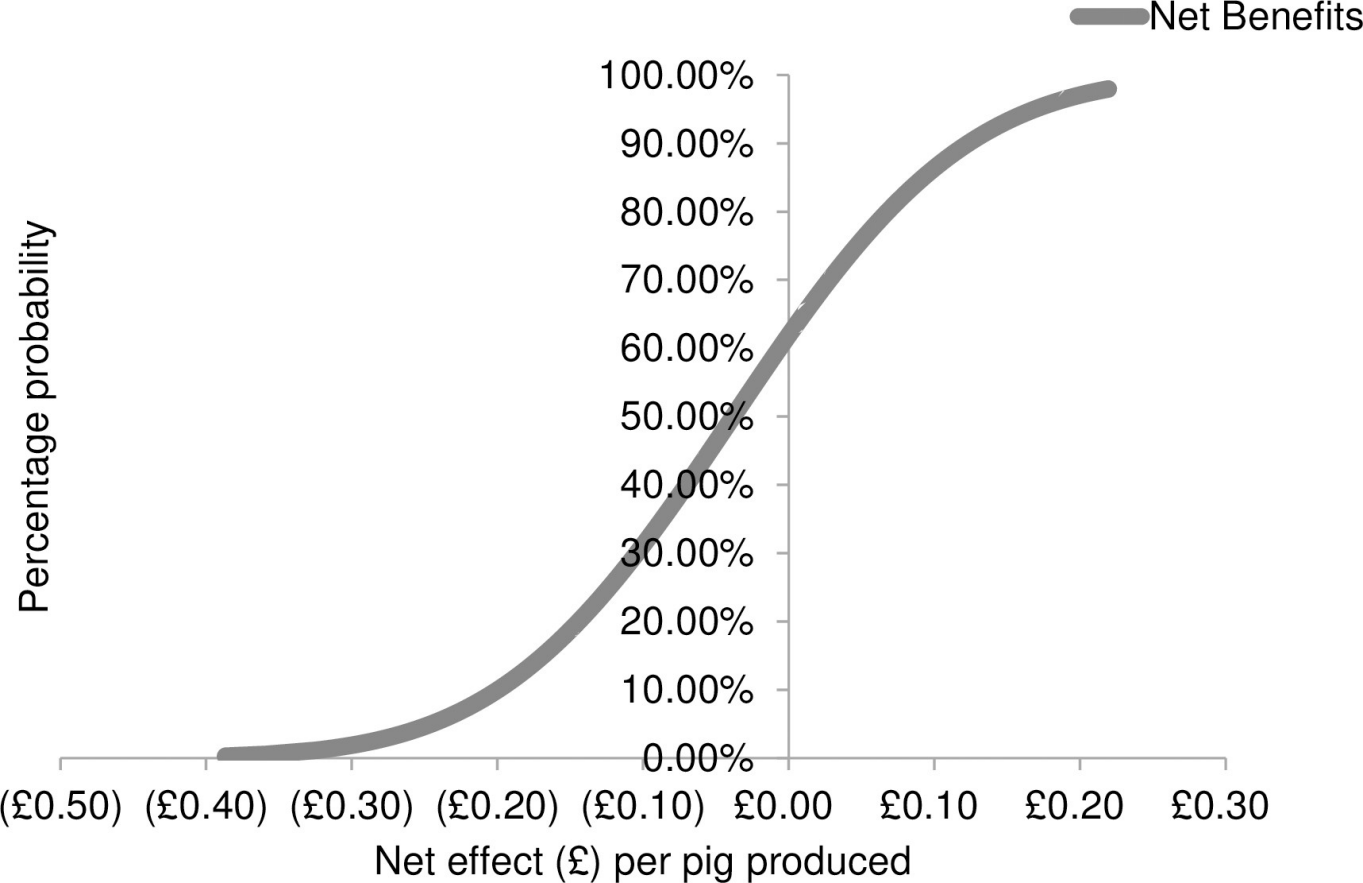
The Cumulative Distribution Function (CDF) for pre-weaning socialisation of litters as modelled through stochastic partial budgeting.

**Fig 2:**
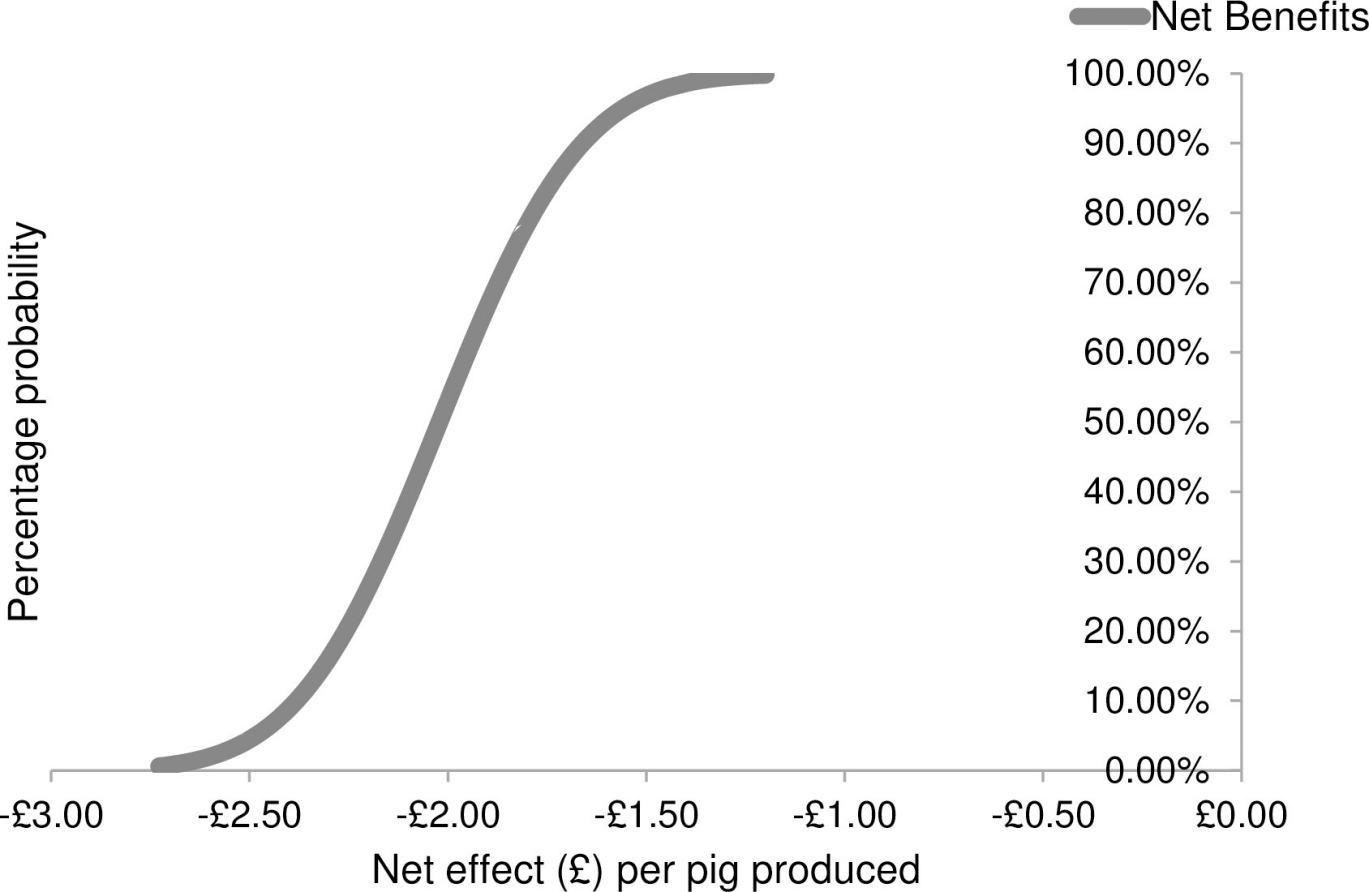
The Cumulative Distribution Function (CDF) for housing pigs in large social groups of 100+ as modelled through stochastic partial budgeting.

**Fig 3:**
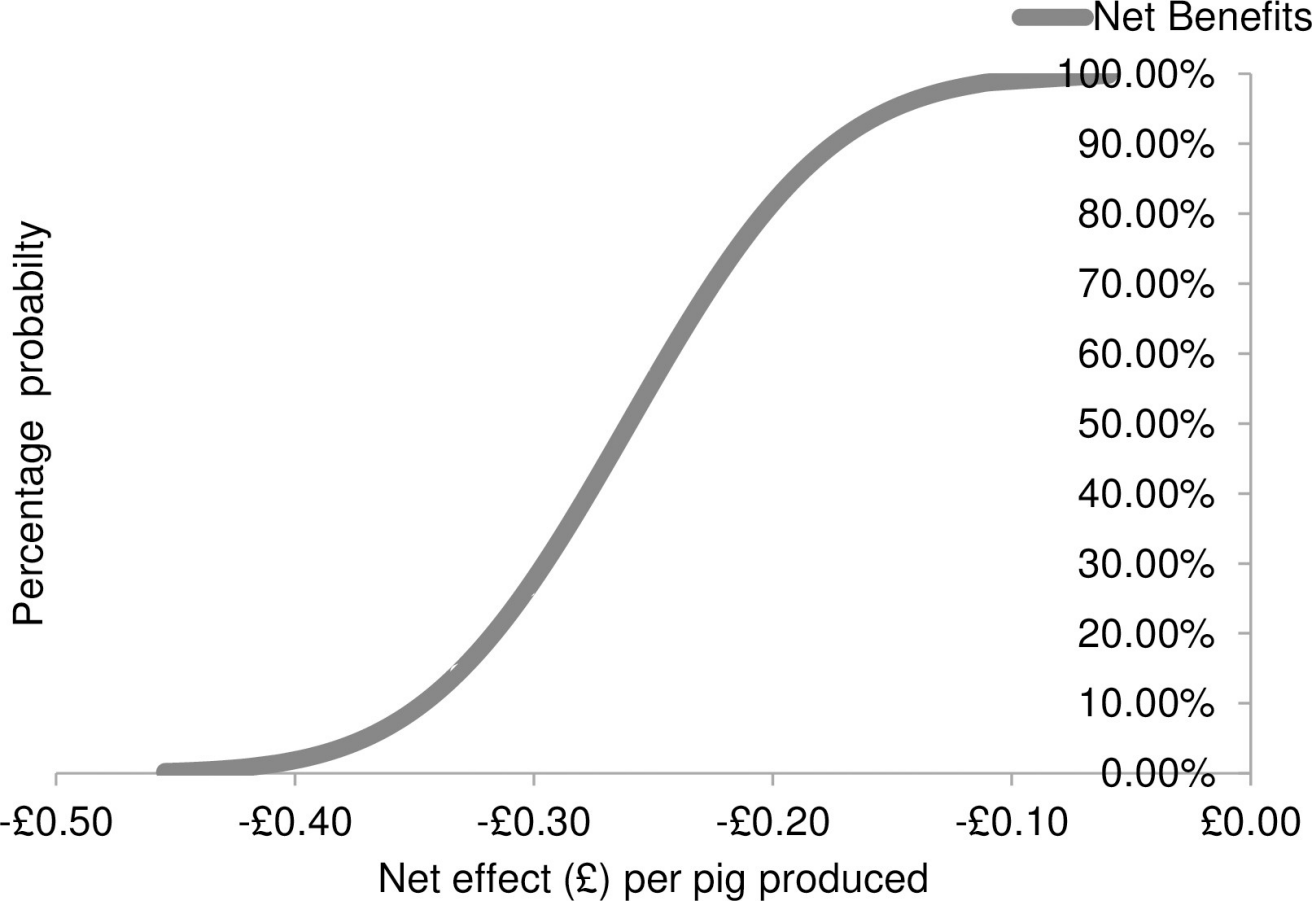
The Cumulative Distribution Function (CDF) for applying synthetic maternal pheromones as modelled through stochastic partial budgeting.

Third, Monte Carlo simulations were used to generate cumulative distributions of the net effect associated with each of the three animal welfare interventions. In particular, the Monte Carlo simulations were used repeatedly to draw 500 random samples from the probability distributions of the net effects. Finally, the cumulative distributions functions were illustrated as graphical forms (see Figs 1–3). In the figures, the cumulative distributions function has probabilities on the vertical axis and associated net effect on the horizontal axis.

## Results

Results revealed that the strategy most likely to be economically viable for farmers was allowing litters to mix prior to weaning (i.e. pre-weaning socialisation). When implementing this strategy, the cumulative distribution function (CDF) line intersects the y-axis at approximately the 62% level (Fig 1). This indicates that farmers are likely to obtain a negative net effect when implementing this strategy 62% of the time. The maximum net cost is £0.39 per pig produced. Farmers are likely to obtain a neutral or positive net effect 38% of the time, with a maximum net benefit of £0.22 per pig produced. When converting existing buildings to house pigs in large social groups, the results indicate that there is 100% probability of negative net effect, and the net effect ranged between -£2.72 and -£1.20 per pig produced (Fig 2). When applying synthetic maternal pheromones there is also 100% probability of obtaining a negative effect; the net effect costs range between-£0.45 and -£0.06 per pig produced (Fig 3).

## Discussion

Animal welfare scientists have identified many changes to animal management, nutrition and genetics which can improve the welfare of livestock, but these interventions are rarely adopted in commercial practice. The economic consequences of animal welfare interventions are a primary determinant of farmers’ willingness to change their current practice [10–12]. Despite this, only a handful of studies have modelled the economic feasibility of specific animal welfare interventions [6, 13–18]. This is in stark contrast with the substantial efforts of animal scientists to identify effective interventions. This study utilised stochastic partial budgeting (SPB) to identify economically feasible animal welfare improvements. Aggression between pigs was used as an example because there is a large literature base from which to draw interventions, and the problem has persisted for decades without resolution. This approach should be extended to other animal welfare issues.

The financial consequences of three aggression control strategies for use when regrouping unfamiliar growing / finishing pigs were estimated, to determine whether they are good investments for farmers. Results are consistent with prior research which indicates that improving animal welfare generally comes at a cost to producers [6, 13–18]. Both exposing pigs to synthetic maternal pheromones and converting existing buildings in order to house pigs in large social groups resulted in 100% probability of obtaining a negative effect. Thus, when implementing these strategies, farmers were likely to obtain a negative net effect 100% of the time. The net effect of exposing pigs to synthetic maternal pheromones was a loss of between £0.06 and £0.45 per pig produced. This loss was primarily determined by the costs of purchasing the commercially available diffusers. Converting existing buildings in order to house pigs in large social groups resulted in a net loss of between £1.20 and £2.72 per pig produced. This loss was primarily associated with the costs of slowed growth rates with increasing group size [59].

Nevertheless, allowing litters to mix prior to weaning (i.e. pre-weaning socialisation) resulted in a neutral or positive net effect 38% of the time, with a maximum net benefit of £0.22 per pig produced. For the majority of surveyed farmers, implementing this strategy required only small labour costs associated with the removal of barriers between adjacent farrowing pens. Pre-weaning socialisation is also the most studied and promising aggression control strategy identified by the academic literature [2]. Therefore, allowing litters to mix prior to weaning should be a fundamental component of campaigns promoting the control of aggression in the industry. However, it is important to note that farmers have expressed multiple concerns about the impact of socialisation on the practical management of sows and piglets, their behaviour, and their growth [32]. Evidence from large scale commercial pig farms suggests that these fears are largely unfounded [36].

A variety of factors influence farmers’ willingness to improve animal welfare in practice (e.g. [7–9]. Peden et al. (2019) modelled 82 UK and Irish pig farmers’ decisions to invest in an aggression control strategy through an economic choice experiment, and identified three independent farmer sub-groups, each with different willingness to pay and preferences for animal welfare and business goals. The first class (18% of respondents) would not invest in an aggression control strategy as they were unlikely to regroup unfamiliar pigs. The second class (32% of respondents) were willing to invest in an aggression control strategy, but were unwilling to pay anything to reduce aggression specifically. They were only interested in the extent to which the strategy improved growth rates. Finally, the third class (50% of respondents) were motivated to reduce aggression for welfare as well as production benefits, and were willing to pay (WTP) £0.11 per pig place (installation cost) and £0.03 per pig produced (running cost) for each 1% reduction in lesions as result of aggression.

In order to assess whether the costs of any aggression control strategy presented in the current study met the demands of farmers in Class 3, their willingness to pay was converted to reflect the costs ‘per pig produced’. As a result, farmers in Class 3 were WTP on average £0.77 per pig produced for a realistic reduction in aggression of 25%. Detailed information on how farmers’ willingness to pay was converted into the ‘per pig produced’ scale is available in S6 File. When the willingness to pay of these farmers was taken into account, both pre-weaning socialisation and synthetic maternal pheromones were likely to be economically viable options, even when the minimum net effect is incurred. Converting existing buildings to increase group size to 100+ pigs remained beyond the realms of farmers’ willingness to pay. Thus, synthetic maternal pheromones should also be included in campaigns promoting the control of aggression in the industry; however, they should be targeted specifically at farmers who are motivated to improve animal welfare for reasons beyond an expected benefit for production.

Converting existing buildings in order to house pigs in large social groups was the least economically viable aggression control strategy analysed in the current study. However, this appears at odds with the findings of Peden et al. (2019a) whereby as many as 45% of 122 surveyed pig farmers reported using large social groups to reduce aggression, and they found them to be moderately useful. The monetary consequences of slowed growth rates were estimated based on the assumption that farmers would increase group size from 50 pigs per pen to 100 pigs. However, large variation in group size, and thus associated costs, exist in the industry. This is illustrated by the finding that the sixteen farmers surveyed in the current study housed growing / finishing pigs in groups of 64 on average (median 39, std 24.9) but this ranged from 15–400. Furthermore, in the study of Peden et al. (2019b) farmers kept growing / finishing pigs in groups of 85.6 on average (median 40, std 121.92) but this ranged from 12–600. The costs of increasing group size rely heavily on initial and target group size; therefore advice should be tailored on an individual farm basis using the methodology described in the current study. Furthermore, although the costs are not justified if converting existing buildings, it is likely that the cost / benefit ratio will be different when building new sheds. When building new sheds, large social groups would be associated with substantial reductions in construction costs related to buying fewer pen divisions and water and feed equipment. Large social groups also allow for automatic sorting technology and other precision livestock developments that enable better individual nutrition and care [68]. Therefore, large groups may be justified if building new sheds, and this might explain the popularity of large groups in the industry.

Farmers’ costs of production vary widely with feed prices, seasons and trading cycles [53]. Pig farmers in the United Kingdom were paid on average £122.07 per pig produced in the first quarter of 2019, but the price fluctuates significantly [53]. For example, the difference in price paid between the best and worst month in 2014 was over £17; in 2016 this was as much as £30; and in 2018 it was £6 [53]. Therefore, pig farmers’ profit margins are small, and highly volatile [69–71] and farmers are likely to be willing to make investments when the industry is performing well but be reluctant to do so when it is not. Thus, the timing of campaigns should take into account how the industry is performing.

The aggression control strategies investigated in the current study were selected based upon published evidence of their effectiveness and the possibility to implement them being in the farmers’ control, which is not the case for strategies that require alternative diet formulations or breeding strategies. Only three aggression control strategies met this criterion. This illustrates that, although many aggression control strategies have been identified by research, very few can be practically implemented and managed by farmers under commercial conditions. Therefore, researchers should concentrate on developing practical solutions. EU legislation requires the control of aggression but does not specify what management interventions are needed to achieve this [72]; all three strategies analysed in the current study could be applied on the large majority of commercial pig farms across the EU.

### Strengths and limitations

This study provides a valuable initial exploration into the costs and benefits of aggression control strategies. However, an important limitation of the current study is that many of the estimates regarding costs and benefits were based on assumptions which contain a high degree of uncertainty. This is due to the limited sample size of farmers included in this survey; the limited availability of monetary cost and benefit data; the large variation in farm size and structure observed in the industry; and the costs and benefits experienced by farmers varying widely over time and country. For example, it was assumed that the lifetime of structural investments would be ten years. However, in reality, the lifetime of structural changes will vary widely depending on the type and quality of construction. Nevertheless, a major strength of the current research is the collaboration with industry stakeholders at all stages. Farmers’ knowledge and experience of pig management mean that the estimations included in the current study are more accurate than could have been estimated by researchers without surveying farmers. Nevertheless, in depth interviews to replace or complement surveys would have been valuable and should be considered in future similar research. Furthermore, uncertainty was accounted for by considering the ‘optimistic’ and ‘pessimistic’ scenarios, and this should be conducted in future economic analysis of welfare interventions.

Farmers who participated were all based in Scotland and it is likely that their estimations are not representative of farmers in other countries [73]. Nevertheless, all farmers who participated in the current study reported housing growing / finishing pigs indoors, as is consistent with the management of growing/ finishing pigs in most major pig producing countries. Furthermore, the farm characteristics and practices regarding pig aggression observed in the current sample of farmers were similar to those of farmers in previous research, who were based across the UK and Ireland [32, 44].

When estimating the monetary benefits of reducing aggression, a linear relationship between financial benefits and the extent to which aggression is reduced was assumed. Specifically, farmers’ estimated the monetary benefits of an ‘optimistic’ reduction in aggression of 50%, and their response was scaled in order to account for the ‘most likely’ scenario of a 25% reduction. The linearity of this relationship has never been studied. Nevertheless, it is not expected that farmers would be able to sensitively take into account any non-linearity in their monetary estimations should it exist.

Farmers are heterogeneous in their willingness to pay for animal welfare, and their motivations for doing so [19]. A major strength of this research is that pairing the information from the SPB with knowledge of the heterogeneity in farmers’ preferences and WTP would allow targeting of advice to classes of farmers most likely to be willing to implement the selected strategies. Quantification of farmer WTP and the heterogeneity in this should be considered in future research of this type.

This research did not consider the role of consumers. Under animal welfare and organic quality assurance schemes, the additional costs of certain animal welfare improvements can be passed to consumers through product labelling [74, 75]. For example, RSPCA assured pigs benefit from freedom of movement during farrowing and increased space allowances, and consumers pay a price premium for these products [76]. However, no quality assurance scheme makes any specific requirements with regards to the control of pig aggression. Therefore, the costs associated with reducing aggression cannot be passed to consumers, and cannot be included in the current economic model as a monetary benefit.

Finally, when using Monte Carlo simulation it is important to account for correlations between parameters [77]. However, we were not able to estimate the correlation matrix due to the nature and availability of sufficient data. For example, because costs and benefits were estimated using a combination of different sources, and because farmers did not provide information on each element.

## Conclusions

The economic feasibility of animal welfare improvements have rarely been estimated despite being fundamental in determining their adoption in practice. This study conducted stochastic partial budgeting of strategies to reduce aggression between pigs, in order to identify economically viable solutions. Results revealed that pre-weaning socialisation is the most economically viable aggression control strategy. The strategy resulted in a neutral or positive net effect 38% of the time. When negative net effects did arise, they were small and within the realms of willingness to pay for a sub-group of farmers with animal welfare goals. Exposing pigs to synthetic maternal pheromones did not improve profitability; nevertheless, the economic costs were also small and within the realms of willingness to pay for a sub-group of farmers with animal welfare goals. The costs of converting existing buildings in order to house pigs in large social groups were high and beyond the realms of farmers’ WTP. The approach adopted in this study, of combining stochastic partial budgeting with willingness to pay from the sector, should be extended to other animal welfare issues.

## Supporting information

S1 File Farmer survey. The survey completed by farmers.
(PDF)

S2 File Conversion into per pig produced. Detailed information on how farmers’ cost estimations were converted into the ‘per pig produced’ scale.
(DOCX)

S3 File Costs of slowed growth rate in large groups. Detailed information on how the costs of lowered growth performance with increasing group size were calculated.
(DOCX)

S4 File Costs of pheromones. Detailed information on how the costs of synthetic maternal pheromones were calculated.
(DOCX)

S5 File Benefits of large social groups. Detailed information on how the benefits of large social groups were calculated.
(DOCX)

S6 File Conversion of farmers’ willingness to pay. Detailed information on how farmers’ willingness to pay was converted to reflect ‘per pig produced’.
(DOCX)
