# Supplementary material for: An estimation of the financial consequences of reducing pig aggression
Source: PLoS One. 2021 May 5;16(5):e0250556. doi: 10.1371/journal.pone.0250556 (PMC8099067; doi:10.1371/journal.pone.0250556)
Supplement: S1 File — The survey completed by farmers. (PDF) [file pone.0250556.s001.pdf]

<u>Estimating the costs and benefits of reducing pig aggression</u>

Researchers have been studying **regrouping aggression between pigs** since the 1970s (aggression that occurs following regrouping of unfamiliar pigs). As a result, several ways of **reducing aggression at regrouping** have been identified. For example, aggression may be reduced by:

- Housing pigs in large social groups (100+)
- Allowing litters to mix prior to weaning

We want to conduct **cost-benefit analyses** to see if these strategies are **financially profitable** for farmers. This cost-benefit analysis involves: (1) calculating the financial **costs** of employing each strategy, (2) calculating the financial **benefits** of employing each strategy, and (3) subtracting the costs from the benefits. If the benefits outweigh the costs, employing that strategy would result in **increased profitability for farmers**.

We would greatly appreciate your help and expertise in <u>estimating the financial costs and benefits of employing aggression control strategies.</u>

What are your current costs per slaughter pig lifetime?

Roughly how many of the following do you have on your farm at any one time?

**Weaners:**		**Growers:**	
**Finishers:**		**Sows:**	

When do you routinely mix unfamiliar pigs on your farm? *Please circle all that apply*

Never	***At finisher***	***Other, please specify:***
At weaning	***At slaughter***	
At grower	***Sows***	

What is your average group size for growing/ finishing pigs?

Do you currently allow different litters to mix <u>prior</u> to weaning (i.e 'co-mingling' or 'socialisation')?

Yes / No

Do you currently apply any chemical additives to feed, water or air e.g. appeasing pheromones, citronella sprays, herbal remedies?

Yes / No

Housing pigs in large social groups	
**Please imagine that you are going to start housing growing/ finishing pigs in groups of 100 or more on your farm**:	
**What <u>initial investments/changes</u> would you need to make?** (e.g. would you need to make any specific changes to your farm?). Please list below.	**How much do you estimate that these changes would cost?** Please make it clear how these costs are described e.g. per pig or per pen
**After the initial investment costs:**	
**Can you envisage any <u>on-going</u> costs associated with housing growing/ finishing pigs in groups of 100 or more?** (e.g. increased labour requirements).	**How much do you think it would cost?** Please make it clear how these costs are described e.g. per pig, per pen or per year

Allowing litters to mix prior to weaning	
**Please imagine that you are going to start allowing litters to mix prior to weaning**:	
**What <u>initial investments/changes</u> would you need to make?** (e.g. would you need to make any specific changes to your farm?). Please list below.	**How much do you estimate that these changes would cost?** Please make it clear how these costs are described e.g. per pig or per pen
**After the initial investment costs:**	
**Can you envisage any <u>on-going</u> costs associated with allowing litters to mix prior to weaning?** (e.g. increased labour requirements).	**How much do you think it would cost?** Please make it clear how these costs are described e.g. per pig or per pen

**Please imagine that you see a reduction in regrouping aggression of 50% in your growers/ finishers. What benefits would you expect to see?** *please circle all relevant responses*	
Improved growth rates	*Improved feed efficiency*
Reduced skin lesions/injury	*Reduced veterinary costs*
Reduced labour requirements	*Improved job satisfaction*
Easier animal handling	*Reduced mortality*
Other, please specify:	

**How much money would you expect to save if you saw a 50% reduction in regrouping aggression in growers/ finishers?** Please make it clear how these costs are described e.g. per pig or per pen

**How are growing / finishing pigs housed on your farm?** *please circle*			
Growers:	*Indoor*	*Outdoor*	*Combined*
Finishers:	*Indoor*	*Outdoor*	*Combined*

**Are you a member of a Quality Assurance Scheme?** *Please circle all that apply*		
No scheme	*Scottish SPC accreditation*	*Genesis Quality*
RSPCA Assured	*Quality Meat Scotland*	*Red Tractor*
Soil Association	*Assured British Pigs*	*Other, please specify:*

**Gender** *(please circle)*	*Male*	*Female*	*Prefer not to say*
**Roughly how many years have you worked with pigs?**			

**Location** *(please circle)*		
England	*Wales*	*Republic of Ireland*
Scotland	*Northern Ireland*	*Other*
