# Supplementary material for: An estimation of the financial consequences of reducing pig aggression
Source: PLoS One. 2021 May 5;16(5):e0250556. doi: 10.1371/journal.pone.0250556 (PMC8099067; doi:10.1371/journal.pone.0250556)
Supplement: S2 File — Detailed information on how farmers’ cost estimations were converted into the ‘per pig produced’ scale. (DOCX) [file pone.0250556.s002.docx]

**S2 File. Detailed information on how farmers’ cost estimations were converted into the ‘per pig produced’ scale**

In order to make the responses of each farmer comparable, their monetary responses were transformed to describe the costs ‘*per pig produced*’ (British Pounds Sterling, GBP). For example, if a farmer specified an *on-going cost* in GBP per pen, this was divided by their group size for growing / finishing pigs (the number of pigs per pen), thus providing a monetary value ‘*per pig produced’*. If a farmer specified an *initial investment cost* in GBP per pen, again, this was divided by their group size for growing / finishing pigs. However, as this *initial investment cost* would last for *many* production cycles, this provided a monetary value ‘*per pig place’*. In order to transform ‘*per pig place*’ values into ‘*per pig produced*’ values, it was assumed that each intervention would have a lifespan of ten years. For housing pigs in large social groups, as described below, it was calculated that each growing and finishing ‘place’ would occupy 144 pigs over the course of this time period. For example, one farmer estimated that it would cost £3.00 *per pig place* to restructure the pens to keep pigs in large social groups, and this value was transformed into ‘*per pig produced’* using the below calculation:

*£3.00 per pig place / 144 pigs over the course of ten years = £0.02 per pig produced.*

It was estimated that 144 pigs would occupy each growing and finishing pig ‘place’ over the course of ten years based on the following assumptions. Pigs enter the grower shed at around 8 weeks old and the finisher shed at around 15 weeks old, where they are housed until slaughter at around 22 weeks old. Therefore, pigs are housed in the growing / finishing facilities for roughly 14 weeks and this is roughly equally split between the grower shed and the finisher shed. Farmers tailor the size of their sow herd to ensure that the weaner, grower and finisher sheds are always occupied other than during the time taken to clean pens between batches. Without accounting for cleaning, each growing shed and each finishing shed has the capacity for roughly 7.4 production cycles per year (52 weeks per year / 7 weeks per production cycle). Over the course of ten years this would be roughly 74 pig production cycles per shed. In order to account for time taken to clean pens in between batches, this was reduced to 72 production cycles per shed over the course of ten years (1 day cleaning per batch x 72 batches = 72 days cleaning over the course of ten years). Both the growing and finishing sheds were accounted for (72 batches per shed over the course of ten years x 2 sheds = 144 pigs produced per place). These assumptions were based on discussions with three senior animal science researchers, all with extensive experience of pig production.

For allowing litters to mix prior to weaning, the initial cost would be incurred in the farrowing house, rather than in the growing / finishing pens. It was estimated that 80 pigs would occupy each farrowing pen place over the course of ten years based on the following assumptions. The majority of sows housed in commercial production systems are moved into farrowing crates or pens roughly 7 days before they are expected to farrow. Sows remain in the farrowing house until their litter is weaned at about four weeks old. Therefore, occupancy is roughly 5 weeks per litter, and without accounting for cleaning, each farrowing pen has the capacity for roughly ten production cycles per year (52 weeks per year / 5 weeks per production cycle). Over the course of ten years this will be roughly 100 production cycles per pen. In order to account for time taken to clean pens in between batches and the fact that sows will farrow on different days during a one week period, this was reduced to 80 production cycles per shed over the course of ten years (7 additional days per batch x 100 batches = 700 additional days over the course of ten years). These assumptions were based on discussions with two senior animal science researchers, both with extensive experience of pig production.

**Limitations:**

When transforming costs ‘per pig place’ into ‘per pig produced’ it was assumed that the lifetime of each intervention would be ten years. For converting existing growing / finishing sheds in order to house pigs in large social groups, it was assumed that 144 pigs would occupy each growing / finishing ‘place’ over the course of this time period. For allowing litters to mix prior to weaning, it was assumed that 80 pigs would occupy each farrowing pen ‘place’ over the course of this time period. These values are highly estimated and in reality, the lifetime of structural changes will vary widely depending on the type and quality of construction.
