# Supplementary material for: An estimation of the financial consequences of reducing pig aggression
Source: PLoS One. 2021 May 5;16(5):e0250556. doi: 10.1371/journal.pone.0250556 (PMC8099067; doi:10.1371/journal.pone.0250556)
Supplement: S3 File — Detailed information on how the costs of lowered growth performance with increasing group size were calculated. (DOCX) [file pone.0250556.s003.docx]

**S3 File. Detailed information on how the costs of lowered growth performance with increasing group size were calculated**

In order to account for the lowered growth performance with increasing group size, a penalty of £1.60 per pig produced was added to the mean scenario, and £1.20 and £2.00 to the ‘minimum’ and ‘maximum’ scenarios respectively. These costs were estimated based on the findings of Turner et al (2003) that average daily gain for a growing pig is 0.65 kg per day minus 0.00048 kg per additional pig (1). To calculate the costs of increasing group size to 100+ pigs, it was assumed that the average group size for growing pigs in the UK industry is 50. This assumption is based on surveying a representative sample of eighty-two UK pig farmers’ and finding that they keep growing pigs in groups of 47 on average (median 39, std 31.6, range 14-200) (2). Based on the study of Turner et al (2003), the average daily gain per pig housed in a group of 50 is approximately 0.63 kg (0.00048 kg per day x 50 pigs = 0.02 kg, 0.65 – 0.02). In comparison, the average daily gain per pig housed in groups of 100 is 0.60 kg (0.00048 kg per day x 100 pigs = 0.05 kg, 0.65 kg – 0.05 kg). Therefore, increasing group size from 50 pigs to 100 pigs would result in a penalty of 0.03 kg per pig per day (0.63 kg – 0.60 kg).

The monetary implications of this growth penalty were estimated based on the following calculations. According to national data obtained by the Agricultural and Horticultural Development Board (AHDB) Pork, in the first quarter of 2019 the average carcass weight of a UK pig (including both standard and non-standard, i.e. RPSCA assured, outdoor born, outdoor bred, pigs) was 85.11 kg (3). Farmers were paid on average 143.42 pence per kg, and thus £122.07 per pig produced (85.11 kg x 143.42 pence per kg) (3). However, ‘carcass weight’ refers to the partially butchered, valuable part of the carcass, and this reflects only approximately 75-76% of the entire animal (4). Therefore, the average liveweight of an entire UK standard pig at slaughter is approximately 112.73 kg (85.11 kg / 75.5% x 100). Based on the assumption that pigs spend 7 weeks (49 days) in the growing stage of production, pigs housed in groups of 100 would be 1.47 kg lighter at slaughter (0.03 kg x 49) than those at an average group size of 50. This reflects a liveweight at slaughter of 111.26 kg (112.73 kg – 1.47kg), a carcass weight of 84.00 kg (111.26 / 100 x 75.5%), and a payment of £120.47 per pig produced (84.00 kg x 143.42 pence per kg). Thus, the cost of reduced growth performance when increasing group size from 50 to 100 pigs is £1.60 per pig produced (£122.07 per pig produced - £120.47 per pig produced). To account for uncertainty in these calculations, the minimum scenario reflects a 25% reduction in the £1.60 cost per pig (75% of £1.60 = £1.20) and the maximum scenario reflects a 25% increase in the £1.60 costs per pig (125% of £1.60 = £2.00).

**Limitations:**

When estimating the monetary costs of reduced growth rate when housing pigs in large social groups, it was assumed that farmers would increase group size from the initial size of 50 pigs. It was necessary to select an initial group size for the purpose of this study, and this was the best estimate based on prior survey research. However, group size varies widely in the industry and the economic consequences would be more accurate if calculations were tailored for individual farms. Furthermore, it was assumed that farmers were paid £122.07 per pig produced. This is the amount farmers were paid in the first quarter of 2019 according to national data (3). However, the price farmers are paid on an individual pig basis fluctuates significantly over time. How the industry is performing will strongly influence the ratio of costs and benefits. The study of Turner et al (2003) was a meta-analysis of a large number of published studies available at that time and no subsequent publications have revised the meta-analysis with new data. Furthermore, the meta-analysis assumes that the impact on average daily gain scales linearly with group size, but this has never been investigated. Lastly it is acknowledged that average daily gains are likely to have increased since 2003 which might have affected the weight gain penalty per additional pig in the group.

**References**

1. Turner SP, Allcroft DJ, Edwards SA. Housing pigs in large social groups: a review of implications for performance and other economic traits. Livestock Production Science. 2003;82(1):39-51.

2. Peden RSE, Akaichi F, Camerlink I, Boyle L, Turner SP. Farmers' willingness to pay for managements strategies to reduce aggression between pigs. PLoS One. 2019.

3. AHDB. Cost of production and net margins. <https://pork.ahdb.org.uk/prices-stats/costings-herd-performance/cost-of-production-and-net-margins/> (accessed 3rd September 2019).

4. AHDB. Factors affecting killing-out percentage <https://pork.ahdb.org.uk/media/273333/afp07_finishing_factors-killing-out_for-web_aw.pdf> (accessed 21st August 2019).
