# Supplementary material for: An estimation of the financial consequences of reducing pig aggression
Source: PLoS One. 2021 May 5;16(5):e0250556. doi: 10.1371/journal.pone.0250556 (PMC8099067; doi:10.1371/journal.pone.0250556)
Supplement: S4 File — Detailed information on how the costs of synthetic maternal pheromones were calculated. (DOCX) [file pone.0250556.s004.docx]

**S4 File. Detailed information on how the costs of synthetic maternal pheromones were calculated**

Synthetic maternal pheromones are commercially available. Therefore, cost-benefit analysis was conducted on the use of synthetic maternal pheromones in accordance with the product instructions and commercially available cost information, and thus did not require the inclusion of synthetic maternal pheromones in the farmer survey. Specifically, diffusers should be placed 1.5m above the ground, one diffuser should be placed every 25m^2^, and the diffusers will release the pheromone for up to 6 weeks following opening. Growing pigs weighing 60kg are provided with 0.55m^2^ (1); therefore one diffuser would account for approximately 45 pigs. Price information for pig appeasing pheromone diffusers was not available for use in this study. Therefore, in order to identify the monetary costs for inclusion in the economic model the costs of purchasing diffusers for companion animals were reviewed: cat diffusers cost £16.99 each (2); dog diffusers cost £13.99 each (3) and general pet diffusers cost £21.00 each (4). Based on the application instructions and price information described above, it was estimated that the cost of purchasing synthetic maternal pheromones would be £0.39 per pig produced (std £0.08, min £0.31 - max £0.47). Specifically: £16.99 / 45 pigs = £0.38 per pig; £13.99 / 45 pigs = £0.31 per pig; £21.00 / 45 pigs = £0.47 per pig; mean = £0.39.

It was assumed that the labour requirements of exposing pigs to synthetic maternal pheromones (i.e. placing diffusers in pens) would be ten minutes per diffuser. This assumption was based on reviewing the available information on diffuser implementation and on discussion with one commercial pig farmer and one senior animal science researcher, both with extensive experience of working with pigs. Thus, a penalty of £0.03 per pig produced was added to the ‘mean’ scenario, and £0.01 and £0.04 was added to the minimum and maximum scenarios respectively. A Grade 2 (standard) agricultural worker is required to be paid a minimum of £6.96 per hour (5). Therefore, it would cost £0.03 per pig produced to install diffusers into pens (£6.96 / 60 minutes x 10 minutes = £1.16 per pen / 45 pigs). The minimum scenario reflects it taking 5 minutes to install each diffuser (£6.96 / 60 minutes x 5 minutes = £0.58 per pen / 45 pigs), and the maximum scenario reflects it taking 15 minutes (£6.96 / 60 minutes x 15 minutes = £1.74 per pen / 45 pigs).

**Limitations:**

When estimating the costs of exposing pigs to synthetic maternal pheromones, the price of pheromones for companion, rather than farm animals was used due to cost information for pig pheromones being unavailable. The production and marketing costs are likely to differ between the agricultural and companion animal industries; therefore some differences may occur in pricing.

**References**

1. Council Directive 2008/120/EC. Laying down minimum standards for the protection of pigs. Official Journal of the European Union.

2. Feliway. Felliway classic diffuser <https://www.feliway.com/uk/Products/FELIWAY-CLASSIC-Diffuser> (accessed 13th August 2019)

3. Adaptil. Adaptil calm diffuser <https://www.adaptil.com/uk/Products/ADAPTIL-Calm-Home-Diffuser> (accessed 13th August 2019)

4. Pet Remedy. Pet Calming Plug in diffuser <http://petremedy.co.uk/product/pet-calming-plug-in-diffuser/> (accessed 13th August 2019)

5. GOV.UK. Agricultural workers' rights <https://www.gov.uk/agricultural-workers-rights/pay-and-overtime> (accessed 20th August 2019)
