# Supplementary material for: An estimation of the financial consequences of reducing pig aggression
Source: PLoS One. 2021 May 5;16(5):e0250556. doi: 10.1371/journal.pone.0250556 (PMC8099067; doi:10.1371/journal.pone.0250556)
Supplement: S5 File — Detailed information on how the benefits of large social groups were calculated. (DOCX) [file pone.0250556.s005.docx]

**S5 File. Detailed information on how the benefits of large social groups were calculated**

Regarding housing pigs in large social groups, an additional benefit was added due to the reduced requirement for gates and partitions which would reduce the time taken to clean pens between batches (1). To estimate these benefits, it was assumed that it would take one stockperson 1 hour to clean two pens sized for 50 pigs each, and 55 minutes to clean one pen sized for 100 pigs. This assumption was based on discussion with one commercial pig farmer and one senior animal science researcher, both with extensive experience of working with pigs. A Grade 2 (standard) agricultural worker is required to be paid a minimum of £6.96 per hour (2). Therefore, it would cost £0.07 per pig place to clean two pens sized for 50 pigs each (£6.96 / 100 pigs) and £0.06 per pig produced to clean one pen sized for 100 pigs (£6.96/60 minutes x 55 minutes = £6.38 / 100 pigs). The saving would therefore be approximately £0.01 per pig produced. For the maximum scenario it was assumed that it would take 45 minutes to clean one pen sized for 100 pigs (£6.96 / 60 minutes x 45 minutes = £5.22 / 100 pigs = £0.05 per pig produced); thus, saving £0.02 per pig produced. For the minimum scenario it was assumed that no time would be saved for cleaning.

**References**

1. Finn J. Pig group size – what is the optimum? . Proceedings of Regional Pig Conferences; Kilkenny, County Longford, Republic of Ireland2004. p. 82–90.

2. GOV.UK. Agricultural workers' rights <https://www.gov.uk/agricultural-workers-rights/pay-and-overtime> (accessed 20th August 2019)
