# Supplementary material for: An estimation of the financial consequences of reducing pig aggression
Source: PLoS One. 2021 May 5;16(5):e0250556. doi: 10.1371/journal.pone.0250556 (PMC8099067; doi:10.1371/journal.pone.0250556)
Supplement: S6 File — Detailed information on how farmers’ willingness to pay was converted to reflect ‘per pig produced’. (DOCX) [file pone.0250556.s006.docx]

**S6 File. Detailed information on how farmers’ willingness to pay was converted to reflect ‘per pig produced’**

Farmers in Class 3 (50% of respondents) were motivated to reduce aggression, and were willing to pay £0.11 per pig place (installation cost) and £0.03 per pig produced (running cost) for each 1% reduction in lesions as result of aggression. In order to convert WTP ‘per pig place’ into WTP ‘per pig produced’, it was assumed that 144 pigs would occupy each growing and finishing ‘place’ over the course of the lifespan of each intervention. Justification for this assumption is described in Appendix B. Based on this assumption, farmers in Class 3 were WTP £0.0008 investment costs per pig produced (£0.11 / 144) and, in total, £0.0308 per pig produced for each 1% reduction in aggression (£0.03 + £0.0008). Thus, farmers in Class 3 were WTP an additional £0.77 per pig produced for a realistic reduction in aggression of 25% (£0.0308 x 25%).
